# Relationship of Neonatal Seizure Burden Before Treatment and Response to Initial Antiseizure Medication

**DOI:** 10.1016/j.jpeds.2024.113957

**Published:** 2024-02-13

**Authors:** Adam L. Numis, Hannah C. Glass, Bryan A. Comstock, Fernando Gonzalez, Nathalie L. Maitre, Shavonne L. Massey, Dennis E. Mayock, Ulrike Mietzsch, Niranjana Natarajan, Gregory M. Sokol, Sonia Bonifacio, Krisa Van Meurs, Cameron Thomas, Kaashif Ahmad, Patrick Heagerty, Sandra E. Juul, Yvonne W. Wu, Courtney J. Wusthoff

**Affiliations:** 1 Department of Neurology and Weill Institute for Neuroscience, University of California San Francisco, San Francisco, CA; 2 Department of Pediatrics; UCSF Benioff Children’s Hospital, University of California San Francisco, San Francisco, CA; 3 Department of Epidemiology & Biostatistics, University of California San Francisco, San Francisco, CA; 4 Department of Biostatistics, University of Washington, Seattle, WA; 5 Department of Pediatrics, Emory University School of Medicine and Children’s Healthcare of Atlanta, Atlanta, GA; 6 Departments of Neurology and Pediatrics, Children’s Hospital of Philadelphia and Perelman School of Medicine, University of Pennsylvania, Philadelphia, PA; 7 Division of Neonatology, Department of Pediatrics, University of Washington School of Medicine, Seattle, WA; 8 Department of Pediatrics, Indiana University School of Medicine, Indianapolis, IN; 9 Division of Pediatric Neurology, Department of Neurology, University of Washington School of Medicine, Seattle, WA; 10 Division of Neonatal and Developmental Medicine, Department of Pediatrics, Stanford University School of Medicine, Stanford, CA; 11 Departments of Neurology & Pediatrics, Stanford University, Stanford, CA; 12 Department of Pediatrics, University of Cincinnati College of Medicine, Cincinnati, OH; 13 Division of Neurology, Cincinnati Children’s Hospital Medical Center, Cincinnati, OH; 14 Pediatrix Medical Group of San Antonio, Children’s Hospital of San Antonio, San Antonio, TX

## Abstract

**Objective:**

To assess among a cohort of neonates with hypoxic–ischemic encephalopathy (HIE) the association of pretreatment maximal hourly seizure burden and total seizure duration with successful response to initial antiseizure medication (ASM).

**Study design:**

This was a retrospective review of data collected from infants enrolled in the HEAL Trial (NCT02811263) between January 25, 2017, and October 9, 2019. We evaluated a cohort of neonates born at ≥36 weeks of gestation with moderate-to-severe HIE who underwent continuous electroencephalogram monitoring and had acute symptomatic seizures. Poisson regression analyzed associations between (1) pretreatment maximal hourly seizure burden, (2) pretreatment total seizure duration, (3) time from first seizure to initial ASM, and (4) successful response to initial ASM.

**Results:**

Among 39 neonates meeting inclusion criteria, greater pretreatment maximal hourly seizure burden was associated with lower chance of successful response to initial ASM (adjusted relative risk for each 5-minute increase in seizure burden 0.83, 95% CI 0.69-0.99). There was no association between pretreatment total seizure duration and chance of successful response. Shorter time-to-treatment was paradoxically associated with lower chance of successful response to treatment, although this difference was small in magnitude (relative risk 1.007, 95% CI 1.003-1.010).

**Conclusions:**

Maximal seizure burden may be more important than other, more commonly used measures in predicting response to acute seizure treatments.

Although most clinicians begin treating neonatal seizures with antiseizure medication (ASM) at the time of diagnosis, controversy persists as to how aggressively to initiate seizure treatment. Although the World Health Organization advises treating all neonatal seizures,^1^ some clinicians reserve treatment only for recurrent seizures or only for “longer” seizures, possibly to avoid exposure to ASM if seizures are few or brief. There also may be a hope to first observe whether seizures remit without intervention. Establishing baseline seizure burden during a period of observation without treatment has even been suggested as a precursor or inclusion criterion for enrollment in clinical trials of seizure treatments in neonates.^2^

Data in critically ill children and adults have shown that delay in treating seizures is associated with a more refractory course. Each minute that a seizure continues before treatment is associated with a lower chance of response to a first- or second-line ASM.^3–5^ Evidence in children and adults suggests that both the time from start of seizure until treatment and also the cumulative seizure burden, or total time of all seizures before treatment, are important factors in determining treatment responsiveness.^6–9^ There is some evidence that suggests time to treatment and pretreatment seizure burden are inversely related to treatment response in neonates,^10–15^ despite the neonatal brain being wired for ongoing excitation even after a single seizure.^16–18^ It remains uncertain in neonates whether successful treatment of seizures is related to the pretreatment duration, burden, or severity of seizures.

For this study, we used continuous electroencephalogram (cEEG) recordings from neonates with neonatal encephalopathy due to hypoxia–ischemia (ie, hypoxic–ischemic encephalopathy [HIE]) and seizures to test the hypothesis that greater seizure burden before treatment is associated with lower chance of seizure cessation with a single loading dose of ASM. Pretreatment seizure burden was measured using 2 common metrics: maximal hourly seizure burden (greatest cumulative seizure duration in any hour before treatment) and total (cumulative) duration of seizures in minutes. As a secondary aim, we assessed the relationship between time to treatment, from seizure onset to initial ASM in minutes, and chance of seizure cessation.

## Methods

This was a secondary analysis of data collected through the HEAL-EEG trial, which has been previously reported.^19^ HEAL-EEG collected digital cEEG recordings from neonates ≥36 weeks of gestation with moderate or severe HIE who received therapeutic hypothermia and who participated in a randomized trial of erythropoietin (Epo) vs placebo for neuroprotection (NCT02811263).^20,21^ All received therapeutic hypothermia initiated within 6 hours of birth. The main trial and cEEG data collection were approved by the institutional review board at each site. Informed, written parental consent was obtained for all participants.

### Participants

The HEAL-EEG study comprised 7 participating HEAL sites that collected cEEG data on a clinical basis according to the American Clinical Neurophysiology Society’s guidelines for neonates with HIE for at least the duration of therapeutic hypothermia (72 hours) and during the return to normothermia.^19^ If neonates in the study died before the end of that window, EEG duration was correspondingly abbreviated. In addition to HEAL trial exclusion criteria, neonates also were excluded if the quality of the cEEG recorded was insufficient for central neurophysiologist review.^20,21^ From the HEAL-EEG cohort, we included neonates with cEEG confirmation of acute symptomatic seizure in this secondary analysis.

### Clinical and Demographic Data

Medical chart review was performed to collect maternal and neonatal demographics and clinical details. Medication administration records were reviewed for timing and dose of ASM. Decisions regarding when to treat seizures and selection of ASM were made by the treating physician for each participant. Randomization to Epo or placebo and the relationship between Epo and seizures has been reported previously, with no significant difference in seizure incidence between the Epo and placebo groups.^19^

### EEG Acquisition and Interpretation

Each site performed cEEG according to American Clinical Neurophysiology Society standards.^22^ EEG was recorded digitally using a minimum of 8 cerebral electrodes, placed using the international 10-20 system modified for the neonate. EEG was acquired using equipment and software in clinical use at the site. Complete digital cEEG files were deidentified and collected for central review by 2 board-certified pediatric clinical neurophysiologists. Although video was recorded at the time of clinical EEG acquisition, video files were not transmitted for central review, to preserve participant privacy.

For central cEEG review, files were read using Persyst software. Montages and review settings were adjusted to neurophysiologist preference. Neurophysiologists were blinded to clinical details of participants, including whether an ASM was administered as well as timing of ASM administration. Each neurophysiologist independently reviewed each EEG to annotate the start and stop time of every seizure. Seizures were defined as sudden, abnormal EEG events characterized by repetitive and evolving discharges, with a minimum voltage of 2 *μ*V peak-to-peak and a minimum duration of 10 seconds.^23^ Where there was a >10% discrepancy regarding total minutes of seizure burden for a participant, consensus review was performed for individual seizure times. Pearson correlation coefficient was used to assess inter-rater reliability on total minutes of seizure burden from independent interpretations before consensus reads. Swimmer plots were generated to graphically represent seizure time series data along with pertinent clinical data.^24^

Pretreatment seizure burden was quantified by 2 measures. Maximal hourly seizure burden (in minutes per hour and % of hour) was the greatest seizure burden in any 60-minute window before initial ASM. Maximal hourly seizure burden was identified using a sliding 60-minute time window for the duration of cEEG recording before initial ASM administration. Total seizure duration, in minutes, was a summation of the total minutes of EEG seizures before initial ASM administration. As a secondary aim, we calculated the total time in minutes between onset of electrographic seizures and time to first loading dose of an ASM.

### Successful Response to Treatment

A successful response to initial ASM administration was defined as a participant having no further seizures present on cEEG from 30 minutes after adequate intravenous loading dose of an ASM until the end of the cEEG recording. Adequate ASM loading dose was defined as phenobarbital ≥20 mg/kg or levetiracetam ≥40 mg/kg.^25^

### Statistical Analyses

Baseline characteristics and measurements were compared between responders and nonresponders to ASM using χ^2^ tests for categorical variables and Wilcoxon rank sum tests for continuous variables. To estimate the adjusted relative risk of maximum hourly seizure burden, total seizure duration, and time between EEG seizure onset and ASM treatment between groups, we used a Poisson regression model with robust SEs. Log binomial models did not converge. The regression model a priori adjusted for treatment group (Epo or placebo) and HIE severity (moderate or severe) with post hoc adjustments for baseline variables that differed between responders and nonresponders to initial ASM treatment. All analyses were conducted using Stata, version 17 and R software, version 4.0.2. For all analyses, 2-sided *P*-values less than .05 were considered significant.

## Results

To reach the prespecified sample size of 150 for HEAL-EEG, cEEGs were collected and reviewed from 185 neonates. Thirty-five participants were excluded for cEEG recording of insufficient quality for central interpretation. Seizures were documented in 46 of these 150 included neonates (31%). Forty-two of 46 (91%) participants had seizures confirmed on cEEG before administration of ASM, and 4 of 46 (9%) had seizures abate before the administration of an ASM. Three of 42 (7%) participants with seizures confirmed by cEEG did not have the dose of ASM documented. Thirty-nine neonates were included in this analysis. Demographic and clinical characteristics are in Table I.Of the 39 neonates with seizures in this analysis, 13 (33%) had a successful response to initial ASM; 26 (67%) did not respond successfully to initial ASM. Responders differed from nonresponders in that they were more likely to have a shorter time from initial ASM loading dose to cEEG end (Table II). There was no difference in time from birth to cEEG start.

**Figure 1.**
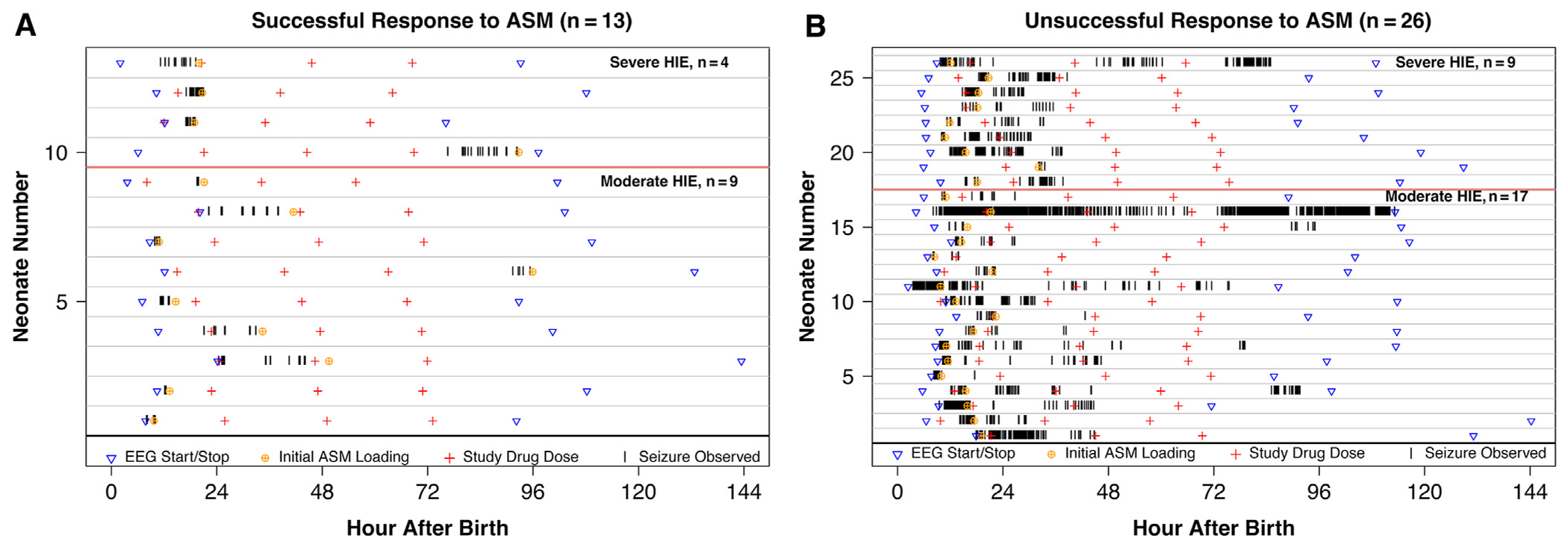
Swimmer plot of 39 neonates with seizures with HIE undergoing therapeutic hypothermia and continuous video-EEG throughout hypothermia and rewarming with **A,** successful response and **B,** unsuccessful (non)-response to initial ASM dosing. *Vertical black lines* indicate that a seizure was observed.

**Figure 2.**
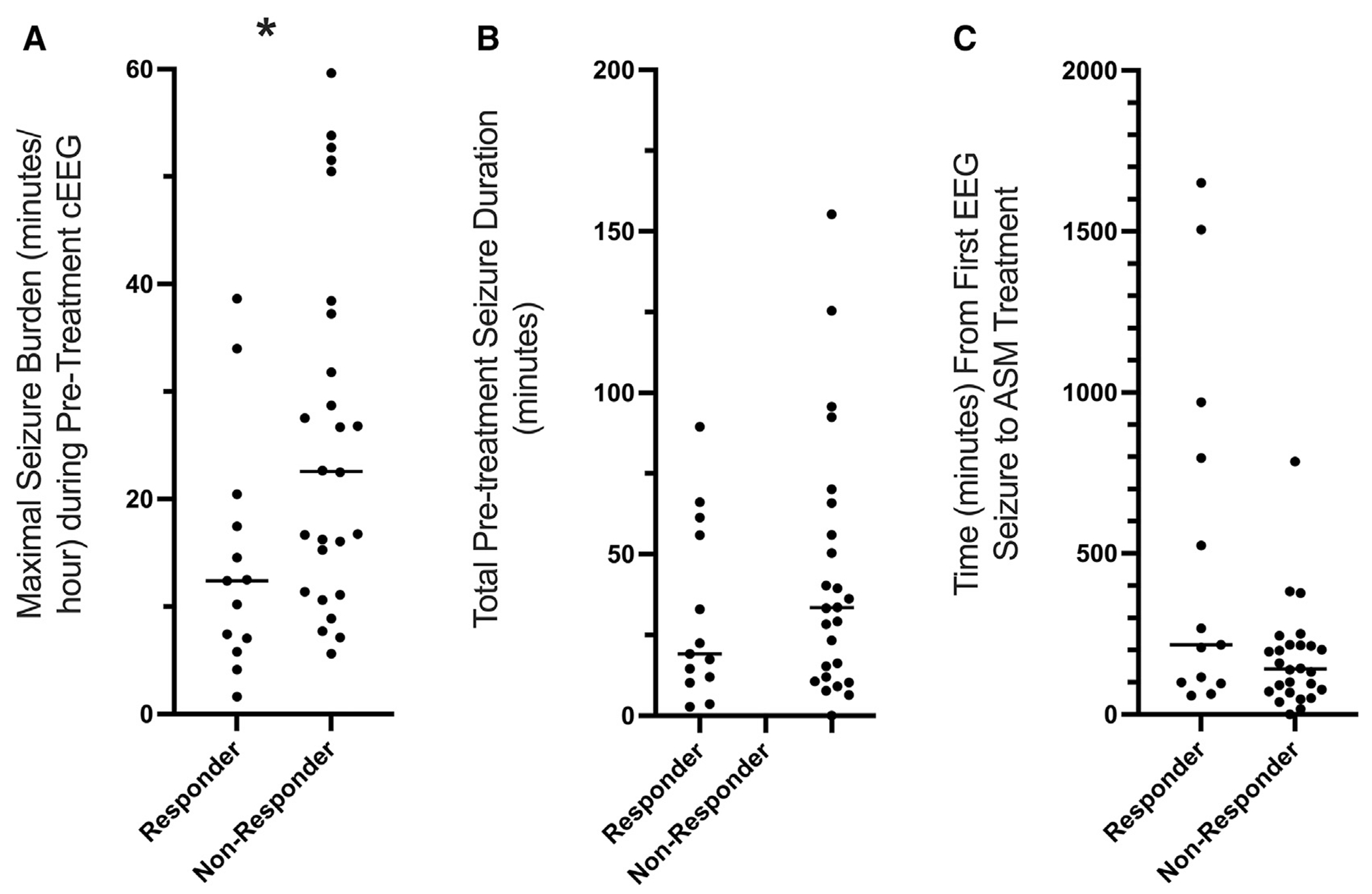
Measures of pretreatment seizure burden among responders to initial ASM treatment and nonresponders. **A,** Maximal hourly seizure burden in minutes/hour among responders and nonresponders. **B,** Total pretreatment seizure duration in minutes among responders and nonresponders. **B,** does not depict one outlier: a nonresponder with total seizure duration of 465 minutes. **C,** Time in minutes from start of first EEG seizure to initial ASM loading dose among responders and nonresponders.

As previously reported, inter-rater reliability for overall seizure burden (minutes of seizure) among neonates with seizures was very high (Pearson r = 0.96).^19^ The characterizations of seizure burden for neonates who did and did not show successful response to initial ASM are shown in Figure 1. The pretreatment maximal hourly seizure burden was lower in those who responded than in those who did not respond to initial ASM loading dose, with a median of 12 min/h maximal seizure burden (20%) in responders (IQR 7-17 min/h), and a median of 23 min/h maximal seizure burden (39%) in nonresponders (IQR 11-37 mins/h) (Table II, Figure 2). There was no difference in pretreatment total seizure duration between responders and nonresponders before the initial ASM loading dose. These findings were unchanged after adjusting for Epo or placebo treatment assignment, HIE severity, and monitoring time after ASM administration (time from initial ASM loading dose to cEEG end). Sensitivity analyses restricted to participants who received phenobarbital as the initial ASM, or calculating logarithmic transformation of the time variables, did not alter these findings.

As a secondary aim, we examined the time, in minutes, from the start of the initial seizure captured on cEEG until the ASM loading dose was given, independent of seizure burden, given data in pediatric cohorts demonstrating an association between longer time-to-treatments and longer convulsion duration.^6,26^ Neonates who showed successful response to initial ASM received first loading dose a median of 217 minutes (IQR 99-796) from the start of first seizure on EEG (Figure 2). In contrast, those who did not show successful response to initial ASM received first loading dose earlier, at a median of 141 minutes (IQR 71-214) from the start of first seizure on EEG. Using Poisson regression, there was a significant albeit modest association of the “time interval from the start of the initial seizure on cEEG until the ASM loading dose was given” with successful response to initial ASM after adjustment for Epo or placebo treatment assignment, HIE severity, and maximal hourly seizure burden before treatment. The relative risk of 1.007 (95% CI 1.003-1.010) indicates that for every 5-minute interval increase in duration from the initial seizure on cEEG to the ASM loading dose there was a 0.7% increase in chance of a successful response.

## Discussion

In this cohort of neonates with moderate or severe HIE who had seizures confirmed on cEEG before receiving a loading dose of ASM, greater maximal hourly seizure burden before treatment was associated with lower chance of successful response to initial treatment. There was no association between cumulative total duration of seizures before treatment and chance of successful response to initial treatment. We did not find evidence of a cumulative seizure threshold beyond which treatment response decreased.

In our analysis, the adjusted relative risk of 0.83, on a multiplicative scale, means that for every extra 5 minutes of peak or maximal hourly seizure burden before the initial ASM was given, there was a 17% decrease in the chance of successful response: For a neonate reaching a maximal seizure burden of 20 minutes of any hour, there would be a less than 50% chance of successful response to the initial load of ASM. Using the 2021 American Clinical Neurophysiology Society definition of status epilepticus,^27^ if the maximal seizure burden reached 12 minutes of any hour, there would be less than a 59% chance of successful response to initial ASM. Therefore, maximal hourly seizure burden may be a key measure of seizure severity and treatment responsiveness. Although we were unable to control for the timing and selection of ASM after neonatal seizures, which were at the discretion of the treating clinicians, our sensitivity analysis supports our conclusion. Future studies adopting real-time monitoring and standardized treatment algorithms may alleviate this potential for bias.^25,28^

The importance of maximal seizure burden is consistent with the known effects of prolonged or repetitive seizures, such as in status epilepticus, in the newborn brain.^15,29^ Prolonged or clustered repetitive seizures involve excessive release of glutamate and other excitatory neurotransmitters with concomitant internalization of inhibitory GABA receptors.^30,31^ Periods of high seizure burden also deplete energy supplies, with a reduction in brain ATP and phosphocreatine after periods of high seizure intensity. When seizure burden is at a high intensity over a prolonged period and without opportunity for recovery, excessive excitatory neurotransmitter release co-occurs with a failure of energy supplies to power neurotransmitter reuptake. These joint changes of excessive synaptic excitation with excitation–inhibition uncoupling may underlie the cellular basis for failure of ASM following a period of high maximal seizure burden. In contrast, the summed total of seizure duration may have less of an impact on response to ASM if seizures are separated by seizure-free intervals that allow for partial energy recovery.

The effect of the maximal seizure burden on physiology may manifest clinically by the consistently demonstrated consequences of neonatal status epilepticus. Although there have been conflicting findings regarding whether the simple presence of seizures is associated with worse outcomes, status epilepticus has repeatedly been shown to increase the risk of adverse outcomes as compared with neonatal seizures of lesser severity.^32–35^ In a highly detailed examination of seizure burden and outcomes after neonatal encephalopathy, Kharoshankaya et al used cEEG to confirm seizure timing and characteristics in 47 neonates.^36^ They found that although the presence of seizures alone was not associated with developmental outcome, greater cumulative seizure duration was associated with abnormal outcome, and greater maximal hourly seizure burden was most strongly associated with abnormal outcome. This is consistent with our finding that of the measures analyzed, maximal hourly seizure burden was the characteristic that was most associated with decreased successful response to initial treatment. Additional follow-up in this cohort will allow for analysis of maximal seizure burden and total seizure duration on developmental outcomes.

Previous studies in neonates have demonstrated mixed evidence that having more seizures before treatment may decrease the chance of successful treatment, with some of these studies limited by a lack of detailed or reproducible cEEG analysis to measure seizure timing and burden. In their study of phenobarbital and phenytoin to treat neonatal seizures, Painter et al included a unique seizure severity index incorporating both the total duration of seizures on EEG and the spatial extent of seizures across EEG channels before treatment. Among the 59 included neonates, a more severe seizure index was associated with a decreased rate of seizure control after either medication was given.^12^ An analysis of the Neonatal Seizure Registry cohorts found that among 534 neonates with seizures, those who had status epilepticus or more frequent seizures were less likely to have successful treatment response after initial ASM, although this study did not include central EEG review and was not able to distinguish whether the timing of high seizure burden was before or after initial ASM.^10^ In contrast, in their pilot trial of bumetanide for neonatal seizure treatment, Soul et al found in post-hoc analysis that greater seizure burden, measured as minutes of seizure per hour, was associated with a more robust response to bumetanide.^15^ Most recently, Pavel et al analyzed data from a cohort of 69 neonates with seizures who received ASM after seizure onset on cEEG.^13^ Subjects were grouped by whether ASM was given within 1 hour of seizure onset on cEEG, given 1-2 hours from seizure onset on cEEG, or given longer than 2 hours after seizure onset. Treatment with ASM within 1 hour of electrographic seizure onset was associated with fewer seizures over the first 24 hours of life. This analysis focused on absolute time to first treatment, in minutes, and did not examine the role of seizure burden. Our results are largely consistent with these previous studies, yet have the additional benefit of precise measures of maximal seizure burden, a metric that could be applied in future studies.

The relationship between absolute time from first seizure to initial treatment and successful treatment response is less clear. Unexpectedly, there was a longer time-to-treatment among those who successfully responded to initial ASM when compared with those who did not. However, the magnitude of this relationship was small and of uncertain clinical significance. Were this finding to be solely a byproduct of the natural history of acute symptomatic seizure decay over time, we would anticipate the effect size to be greater. Alternatively, this finding may be related to the very large range of times-to-treatment among our cohort (with notable outliers). It is also possible this finding reflects another consequence of high peak seizure burdencthose neonates with greatest pretreatment maximal hourly seizure burden, including those with status epilepticus, may have been more rapidly diagnosed, and thus treated more quickly than neonates with isolated, shorter seizures. We could not control for the frequency of cEEG evaluations at each site, which could also influence this finding. Real-time monitoring supported by computational trends and seizure detection algorithms is an area of ongoing work that may facilitate more consistent practice in the future.^37^ Our results differ from the previous work of Pavel et al, which showed time to seizure treatment was associated with reduced overall seizure burden in the first 24 hours.^13^ We do not suggest delaying initiation of ASM after seizures are identified. Rather, early treatment remains a logical strategy, as ASM treatment initiated before seizures have reached a high maximal hourly seizure burden may be more likely to succeed than if treatment is deferred until seizure burden is greater.

Strengths of this study include the prospective collection of cEEG from a multicenter cohort and the meticulous annotation of seizures on cEEG by 2 independent neurophysiologists with high inter-rater agreement to identify seizure timing and burden. This study focused on neonates born at or near term with suspected HIE. Although this allowed for fewer potential confounders related to seizure etiology, it also limited the generalizability of our results when considering neonates born prematurely or with other seizure etiologies. Although our analysis included neonates from multiple sites, our findings may nonetheless be limited by our sample size. With a larger sample size, there may have been a significant association between total seizure duration and response to initial treatment, although it is less likely this would eclipse the association between maximal hourly seizure burden and initial response to treatment. Another limitation of this secondary analysis is that the balance of uncharacterized maternal or neonatal characteristics obtained with randomization in the larger HEAL cohort could be lost, with subsequent confounding. Studies designed to address specifically the association of maximal hourly seizure burden with treatment responsiveness are needed.

In this analysis of cEEG-defined seizure timing and burden in neonates with suspected HIE, we found that greater maximal hourly seizure burden was associated with a lower chance of successful response to initial ASM. Total cumulative seizure duration was not associated with initial treatment response, and shorter time-to-initial treatment was paradoxically associated with lower chance of successful initial treatment, although this relationship was of very small magnitude. Our findings suggest that risks associated with neonatal seizures may be better understood when considering measures of seizure burden than merely the presence or absence of seizures. Maximal seizure burden may better parallel the underlying disruption of neonatal physiology. These results may explain why previous studies have found conflicting results regarding how the time-to-treatment or the cumulative duration of seizures relate to outcomes. Those measures may be less successful in capturing the key feature of seizure burden. Future studies of neonatal seizures should include maximal hourly seizure burden as a core measure to better understand how seizures may truly affect neonatal outcomes. Rather than simple seizure counts, EEG software for automated analysis and clinical neurophysiology reports should include this measure. Maximal hourly seizure burden should be considered a key measure of neonatal seizure severity for both clinical management and future neurodevelopmental outcomes research.

## Figures and Tables

**Table I.** Demographic and clinical characteristics of included neonates

Characteristics	Total n = 39	Successful response to initial ASM n = 13	Inadequate response to initial ASM n = 26	*P* value*
Maternal characteristics, No. (%)				
Age, y, mean (SD)	29.7 (6.8)	31.0 (7.9)	29.0 (6.2)	.60
Education, high school or less	18 (46%)	7(54%)	11(42%)	.57
Parity of 1, including trial infant, No. (%)	23 (59%)	5 (38%)	18 (69%)	.07
Pregnancy and delivery complications, No. (%)				
Maternal chorioamnionitis or fever	7 (18%)	1 (8%)	6 (23%)	.24
Maternal pre-eclampsia or eclampsia	5 (13%)	2 (15%)	3 (12%)	.74
Gestational diabetes	2 (5%)	1(8%)	1(4%)	.61
Maternal obesity (BMI >30)	8 (21%)	4 (31%)	4 (15%)	.26
Sentinel event^†^	11 (28%)	6 (46%)	5 (19%)	.08
Cesarean delivery	26 (67%)	7 (54%)	19 (73%)	.48
Infant characteristics				
Female, No. (%)	18 (46%)	7 (54%)	11 (42%)	.50
Birth weight, g, mean (SD)	3540 (603)	3611 (834)	3503 (462)	.98
Gestational age, wk, mean (SD)	39.6 (1.3)	39.4 (1.5)	39.7 (1.2)	.61
5-minute Apgar score, median (IQR)	3(1-4)	3(1-4)	3(1-4)	.84
Lowest pH,^‡^ mean (SD)	6.9 (0.17)	6.9 (0.20)	6.9 (0.16)	.51
Worst base deficit,^‡^ mean (SD)	–19.3 (5.9)	–17.6 (6.8)	–20.1 (5.4)	.25
Severe encephalopathy,^§^ No. (%)	13 (33%)	4 (31%)	9 (35%)	.81
Erythropoietin treatment arm, No. (%)	20 (51%)	5 (38%)	15 (58%)	.26
EEG and loading ASM				
Time from birth to cEEG start, h, median (IQR)	8.4(6.2-10.4)	10.3(7.0-12.1)	7.6(6.2-9.4)	.20
Time from ASM loading dose to cEEG end, h, median (IQR)	86.3(73-97)	78.2(62-87)	93.7(78-98)	.02
Time from cEEG start to end, h, median (IQR)	97.9 (85-105)	91.2 (86-98)	99.8 (85-105)	.44
Phenobarbital, No. (%)	36 (92%)	12 (92%)	24 (92%)	1.0
Levetiracetam, No (%)	3 (8%)	1 (8%)	2 (8%)	

*BMI,* body mass index.

* *P* values calculated using χ^2^ tests for categorical variables and Wilcoxon rank sum tests for continuous variables. Bolded values highlight statistically significant associations.

† Sentinel events are defined as placental abruption, shoulder dystocia, uterine rupture, or prolapsed cord.

‡ Lowest pH and worst base deficit among cord arterial, cord venous, and arterial blood gas samples taken before 60 minutes of age.

§ Severe encephalopathy as defined by modified Sarnat score.

**Table II.** Seizure burden measures and response to initial ASM

Seizure burden	Total n=39	Successful response to initial ASM n = 13	Inadequate response to initial ASM n = 26	RR (95% CI)*	*P* value	aRR (95% CI)^†^	*P* value
All participants							
Maximal hourly seizure burden during pretreatment cEEG, min/h, median (IQR)	17 (10-32)	12 (7-17)	23 (11-37)	0.80 (0.64-0.99)	.04	0.83 (0.69-0.99)	**.04**
Total pretreatment seizure duration, min, median (IQR) Phenobarbital only	29 (12-61)	19 (12-56)	33 (12-66)	0.96 (0.91-1.02)	.21	0.95 (0.89-1.01)	.11
Phenobarbital only							
No.(%) with data	36 (92)	12 (92)	24 (92)				
Maximal hourly seizure burden during pretreatment cEEG, min/h, median (IQR)	16 (10-27)	11 (6-16)	20 (11-30)	0.73 (0.56-0.96)	**.03**	0.78 (0.61-0.99)	**.04**
Total pretreatment seizure duration, min, median (IQR)	26(11-53)	18(11-47)	31(11-53)	0.96(0.89-1.04)	.32	0.95(0.88-1.02)	.18

*aRR,* adjusted relative risk; *RR*, relative risk.

Bolded values highlight statistically significant associations.

* RRs and *P* values based upon Poisson regression model for each 5-minute interval increase in pretreatment maximal hourly seizure burden or total seizure duration.

† RRs and *P* values based upon Poisson regression model for each 5-minute interval increase in pretreatment maximal hourly seizure burden or total seizure duration and adjusts for treatment, HIE severity, and time (hours) from initial ASM loading dose and cEEG end.

## Data Availability

Data sharing statement available at www.jpeds.com.

## Abbreviations

ASM: Antiseizure medication
cEEG: Continuous electroencephalogram
Epo: Erythropoietin
HIE: Hypoxic–ischemic encephalopathy
